# HDAC2 deregulation in tumorigenesis is causally connected to repression of immune modulation and defense escape

**DOI:** 10.18632/oncotarget.2816

**Published:** 2014-11-25

**Authors:** Mariarosaria Conte, Carmela Dell'Aversana, Rosaria Benedetti, Francesca Petraglia, Annamaria Carissimo, Valeria Belsito Petrizzi, Alfonso Maria D'Arco, Ciro Abbondanza, Angela Nebbioso, Lucia Altucci

**Affiliations:** ^1^ Department of Biochemistry, Biophysics and General Pathology, Seconda Università degli Studi di Napoli, vico L. De Crecchio, Naples, IT; ^2^ Institute of Genetics and Biophysics, IGB ‘Adriano Buzzati-Traverso’, Via P. Castellino, Naples, IT; ^3^ Division of Onco-Hematology, Umberto I Hospital, via S. Francesco, Nocera Inferiore (SA), IT

**Keywords:** HDAC2, leukemia, HDAC inhibitors, MHC class II

## Abstract

Histone deacetylase 2 (HDAC2) is overexpressed or mutated in several disorders such as hematological cancers, and plays a critical role in transcriptional regulation, cell cycle progression and developmental processes. Here, we performed comparative transcriptome analyses in acute myeloid leukemia to investigate the biological implications of HDAC2 silencing versus its enzymatic inhibition using epigenetic-based drug(s). By gene expression analysis of HDAC2-silenced vs wild-type cells, we found that HDAC2 has a specific role in leukemogenesis. Gene expression profiling of U937 cell line with or without treatment of the well-known HDAC inhibitor vorinostat (SAHA) identifies and characterizes several gene clusters where inhibition of HDAC2 ‘mimics’ its silencing, as well as those where HDAC2 is selectively and exclusively regulated by HDAC2 protein expression levels. These findings may represent an important tool for better understanding the mechanisms underpinning immune regulation, particularly in the study of major histocompatibility complex class II genes.

## INTRODUCTION

Acute myeloid leukemia (AML) is associated with a distinct group of clonal hematopoietic stem cell disorders in which both failure to differentiate and over-proliferation in the stem cell compartments result in accumulation of non-functional immature cells termed ‘myeloblasts’. Better insights into the genetic background of AML are currently leading to a wide array of so-called ‘targeted therapies’, many of which are in clinical development. The AML classification system has evolved from being morphologic- to cytogenetic/genetic-based, reflecting the recognition of the importance of subtype-specific biology. Recently, several molecular-based prognostic factors have been described, although the impact of such findings on treatment decisions remains unclear [1]. Standard treatment of AML results in a median survival of approximately one year [2] and outcomes of myelodysplastic syndromes likewise remain poor. Innovative strategies are therefore urgently needed. The increasing understanding of AML biology has led to the introduction of many novel anti-AML drugs [3]. Both hematological and solid neoplasms, such as breast cancer, may be caused by alterations in the balance between histone acetyltransferases (HATs) and histone deacetylases (HDACs)[4]. Aberrant epigenetic modulations, including deregulation of DNA methylation and other post-translational histone modifications such as acetylation [5], together with genetic mutations, are causally linked to cancer. Acetylated histones are involved in an epigenetic mechanism marking transcriptionally active regions of chromatin [6]. Specifically, HATs and HDACs differently regulate protein acetylation levels by modulating gene expression and cellular signals. HATs catalyze the transfer of acetyl groups from acetyl-CoA to the ε-NH_2_ group of lysine residue side chains. In contrast, HDACs contain a highly conserved deacetylase domain, which spans 300 amino acid residues and catalyzes hydrolytic release of the acetyl group [7]. Hence, acetylation-dependent regulatory pathways, in cooperation with additional post-translational modifications, are key homeostasis determinants. In mammals, 18 HDACs have been identified and grouped into four classes. The class I enzymes HDAC1 and HDAC2 are close homologs of yeast Rpd3, which is the most important HDAC regulating total levels of histone acetylation in yeast [8]. HDAC3 and HDAC8 also belong to the class I HDAC family. Class IIa (HDAC4, HDAC5, HDAC7, HDAC9) and IIb (HDAC6, HDAC10) family members are related to the *Saccharomyces cerevisiae* HDAC Hda1p. HDAC11 shares some sequence homology with class IIa and IIb HDACs and is the only member of class IV. Class III HDACs, the mammalian sirtuins (SIRT1–7), are homologs of *S. cerevisiae* silent information regulator 2 (Sir2p). While class I, II and IV HDACs use Zn^2+^for catalysis, class III HDACs use NAD^+^[9]. Structural homology and common catalytic mechanism(s) can be considered as a functional redundancy of HDACs [10]. However, many important physiological functions, such as growth, differentiation, and reactions to external and internal stimuli, may be crucially controlled by a single HDAC. For example, gene expression analyses in brain and cardiac tissues have shown that, despite sharing 80% sequence homology, HDAC1 and HDAC2 affect different sets of target genes. Specifically, HDAC1 and HDAC2 are together involved in early synaptogenesis, whereas HDAC2 has a wide-ranging effect on synaptic transmission in mature neurons [11]. Aberrant expression of HDAC2 has been identified in dystrophic muscles and chronically inflamed tissues [12], as well as in prostate, ovarian, endometrial and gastric cancer. HDAC2 expression and activity are both regulated at transcriptional, post-transcriptional and post-translational levels. HDAC2 occupies the promoter regions of p21 and p57 genes, indicating that regulation of their expression levels controls cell cycle progression. In addition, both HDAC1 and HDAC2 promote G1-S phase transition by inhibiting expression of p21 and p57 [13]. Furthermore, HDAC2 and N-Myc decrease p53 phosphorylation at serine 46, repressing gene transcription of tumor protein 53-induced nuclear protein 1 [14]. HDAC2 is crucial for embryonic development, affects cytokine signaling involved in immune responses, and is often highly up-regulated in solid and hematological tumors [12]. DNA damage is induced during tumor evolution, and HDAC2 is overexpressed in many cancers promoting the effective repair of DNA and regulating histone acetylation, including acetylation of histone H4 on lysine 16. This particular histone modification shows a biphasic response to DNA damage as expression levels are initially reduced, but increase in the long term due to DNA repair. Indeed, replication stress produces an increase in the expression of histone H4 acetylated on lysine 16 [15]. Nevertheless, transformed cells lacking HDAC2 as a result of somatic mutations were recently described [16]. Studies suggest that both individual and specific groups of HDAC enzymes may be associated with certain cancers, and inhibition of HDACs could translate into therapeutic benefit in malignancies. Furthermore, HDAC inhibitors (HDACi) can also be used as sensitization agents in chemotherapy or hormonal intervention [17]. HDACi have been shown to induce cell cycle arrest, differentiation and chromatin de-condensation, to inhibit angiogenesis, and to induce apoptosis [18]. HDACi are classified into six groups according to their chemical structure, and at least 12 are currently in clinical trials [19] [20] [21]. To date, the US Food and Drug Administration (FDA) has approved two HDACi, vorinostat (suberoylanilide hydroxamic acid or SAHA, Zolinza^®^) and romidepsin (FK228, depsipeptide, Istodax^®^) for the second-line treatment of cutaneous T-cell lymphoma. Another widely studied HDACi, entinostat (MS-275), is currently in clinical trials for treatment of Hodgkin's lymphoma and advanced breast cancer. HDACi are also associated with immune-modulatory effects, and much attention is being focused on antigen-presenting cells, which are key regulators of immune activation. The epigenetic silencing of immune genes in cancer may result in a lower checkpoint control and thus in cancer advancement. Increased immune gene repression has been associated with HDAC overexpression. The first study describing the activation of silenced major histocompatibility complex (MHC) genes in many tumor cells was performed with the HDACi Tricostatin A (TSA) [22]. *In vitro* treatment with HDACi can alter the acetylated state of chromatin and trigger the transcription of silenced genes, including MHC class II genes [23]. Systemic treatments with HDACi could potentially enhance host immune responses by correcting the negative effects of cancer cells on host immunity. Recent studies revealed that HDACi-treated tumor cells are capable of activating both innate and adaptive immune responses *in vivo* [24]. Although tumor suppressor and immune genes are often silenced by HDACs in cancer cells, the mechanisms leading to epigenetic silencing are still not well understood. In particular, HDAC2 was reported to inhibit transcription of the *CIITA* gene and expression of MHC class II genes in human cervical cancer cell lines [25]. MHC class II genes are required for the recognition of tumor cells by CD4^+^ T cells, and antigen presentation via MHC class II is critical for activation of adaptive immune responses. HDACi may be used to modify immunity through multiple host and tumor pathways to improve the efficacy of antitumor therapy. Several HDACi are effective in selected immune disease models. For example, treatment with SAHA inhibits TNF-alpha and IL-6 production by stimulating mesangial cells *in vitro,* and blocks renal disease progression in a murine model of systemic lupus erythematosus [26]. A well as elucidating the role of HDAC2 in cellular fate, these findings indicate that HDAC2 is a therapeutically important target that can be controlled pharmacologically. Although it is clear that inhibition of individual HDACs may elicit distinct functions and impact on the biological effects of HDACi, a systematic approach to understand the role of specific HDAC silencing compared to the inhibitory effect of HDACi has not yet been undertaken.

Here, we show that HDAC2 silencing induces modulation of gene expression leading to strong transcriptional activation. The lack of HDAC2 stably determines chromatin changes and a different acetylation state by promoting the transcriptional activation of specific target genes. Furthermore, inhibition of HDAC2 leads to the expression of genes involved in activation of immune responses, such as genes in the human leukocyte antigen (HLA) family [27], required in the effector stages of antitumor immunity. Such active immunoregulation may increase the efficacy of antitumor therapies. Interestingly, by treating wild-type and HDAC2-silenced U937 AML cells for 24 hours with or without SAHA we identified: a) a subset of 163 genes specifically dependent on HDAC2 silencing but not on its inhibition; b) a subset of 582 genes specifically regulated by SAHA only in absence of HDAC2; c) 466 genes exclusively regulated by SAHA, where HDAC2 inhibition/expression is dispensable; d) 28 genes regulated by both SAHA and HDAC2 silencing together; e) 66 genes regulated by SAHA or by HDAC2 silencing; f) 106 genes both regulated by HDAC2 inhibition and by SAHA alone; g) 1620 genes regulated by SAHA only if HDAC2 is expressed.

## RESULTS

### Selective HDAC2 silencing displays anti-proliferative effects in both leukemia and breast cancer

**Figure 1:**
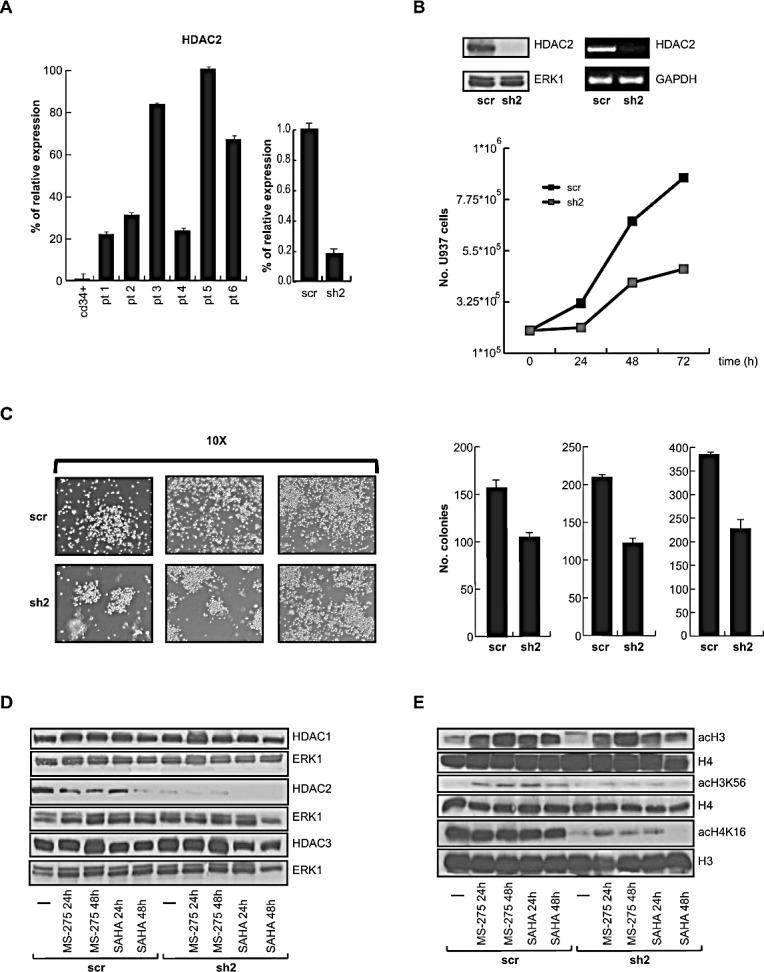
Selective HDAC2 silencing in U937 cells A. Left: Real-time PCR performed on *HDAC2* gene in 6 AML patients. CD34+ cells were used as further control. Right*:* Real-time PCR on sh2 and scr clones. Data show mean values from three parallel experiments with error bars showing standard deviations above each column. B. Upper: Western blot of HDAC2 in sh2 and scr clones. Normalization was performed with ERK1. Semi-quantitative PCR of sh2 and scr clones. Lower*:* Proliferation rate in sh2 and scr clones after 24, 48 and 72 hours. C. Left panel: Colony formation assay on sh2 and scr clones after 14 days of culture at 37°C and 5% CO2. Right panel: Number of colonies in sh2 and scr clones. D. HDAC1, HDAC2 and HDAC3 protein expression levels in sh2 and scr clones at 24 and 48 hours with and without treatment with HDACi MS-275 and SAHA used at a final concentration of 5 μM. Normalization was performed with ERK1. E. Western blot analysis of acetylated histones H3, H3K56, H4 and H4K16 at 24 and 48 hours in sh2 and scr clones with and without treatment with HDACi MS-275 and SAHA both used at a final concentration of 5 μM. Normalization was performed using total histone H3 and H4.

**Figure 2:**
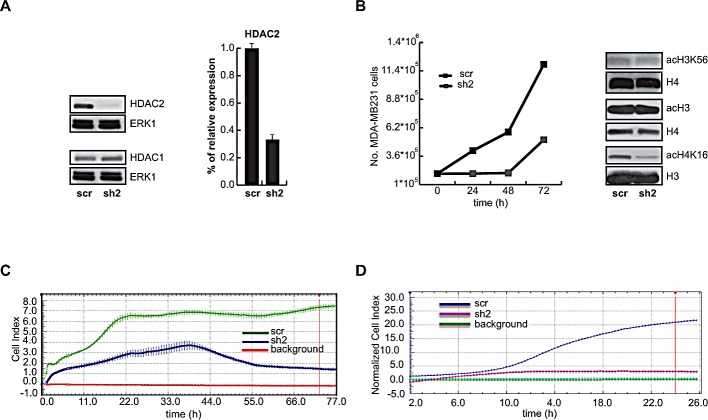
HDAC2 silencing in MDA-MB-231breast cancer cells A. Western blot assays of HDAC1 and 2, and real-time PCR for HDAC2 silencing validation. B. Left: Proliferation curve at 24, 48 and 72 hours for sh2 and scr clones. Right: Western blot of acetylated histones H3, H3K56 and H4K16. ERK1 was used for normalization C. Proliferation curve relative to MDA-MB231 cell line at longer times, showing scr clone (green), sh2 clone (blue) and background (red). D. Migration curve at 24 hours in MDA-MB231 cell line, showing scr clone (blue), sh2 clone (magenta) and background (green).

*HDAC2* gene is strongly upregulated in primary human AML. Quantitative PCR (q-PCR) analysis of cells derived from six AML patients showed a higher HDAC2 expression as compared to normal expression levels in CD34+ myeloid progenitors (Figure 1A and Table 1). In order to investigate the role of deregulation in AML, HDAC2 silencing in U937 AML cell line was performed. More than 100 U937 HDAC2-silenced (sh2) clones were tested for HDAC2 expression. The results clearly indicate a decrease of about 80% in expression of HDAC2 as shown, representatively, in one of the selected sh2 clones by q-PCR (Figure 1A), Western blot and semi-quantitative PCR (Figure 1B). When proliferation rate was assessed, the intrinsic proliferation level in sh2 clone was significantly lower than in scramble (scr) cells mimicking wild-type HDAC2 expression (Figure 1B, bottom). In addition, a colony formation assay was performed in sh2 and scr U937 cells (Figure 1C), corroborating the finding that HDAC2 silencing reduces proliferation and colony formation in leukemia cells. The fact that HDAC1 and HDAC3 protein expression levels remained unchanged with or without treatment using the HDACi MS-275 or SAHA demonstrates both the selectivity of HDAC2 silencing and the specific correlation of the effects observed with the reduction in HDAC2 expression (Figure 1D). To characterize the effects of HDAC2 silencing on chromatin, histone H3 and H4 acetylation levels were tested. Although no differences in either total H3 or H3K56 acetylation levels were detected between scr and sh2 cells with or without treatment using MS-275 or SAHA, acetylation of H4K16 drastically decreased in the sh2 clone (Figure 1E), suggesting that the so-called ‘epigenetic hallmark’ of cancer [28] might be decreased and possibly account for the reduced proliferation of tumor cells. Accordingly, when HDAC2 silencing was induced in MDA-MB231 breast cancer cells using a different sh2 target sequence and a different expression vector (Figure 2A), we obtained similar levels of H3 and H3K56 acetylation and a similar decrease in acetylation levels of H4K16 (Figure 2B). Again in MDA-MB231 cells, both reduction in proliferation rate (Figure 2B and 2C) and (in this case) inhibition of migration potential in real-time curves were detected (Figure 2D). When HDACi response rate was assessed, we did not detect any major difference between sh2 and scr cells, likely suggesting that the presence of all the other HDACs is still sufficient for HDACi treatment response (Supplementary Figure 1A-D).

Taken together, our results show that HDAC2 silencing reduces proliferation and migration of hematopoietic and solid cancer cells without altering HDACi anticancer effects in terms of apoptotic response in these settings.

**Table 1:** Features of AML patients

Pt no.	Age	Diagnosis	FAB	Cytogenetics	Phenotype
1	70	AML	/	46, XY	CD13+, CD34+, CD33+, CD45+-, CD117+-
2	64	AML	M1	/	(gate 90%), CD13+, CD33+, CD19+, TdT+, CD117+, MPO+, CD22cy
3	61	AML	M1	complex	CD13+-, CD33+, CD117+-, CD14+-, CD34+-, CD45+-
4	/	AML	/	/	CD34+, DR+, CD13+, CD33+, CD117+, MPO+, CD38+, CD2+-, CD7+-
5	/	AML	M4	46, XX	(gate 80%), CD34+, CD33+, CD13+, HLA-DR+, CD11b+, CD45RA+, CD71+, CD11c+, CD25+, CD117+, CD64+, CD4+
6	70	suspected monocytosis	M4	46, XX	(gate 33%), CD34+, CD117+, CD13+, CD45RO+, CD33+-, HLA-DR+-, MPO+

### Gene expression profiling identifies a cluster of 269 genes selectively modulated by HDAC2 silencing in leukemia cells

**Figure 3:**
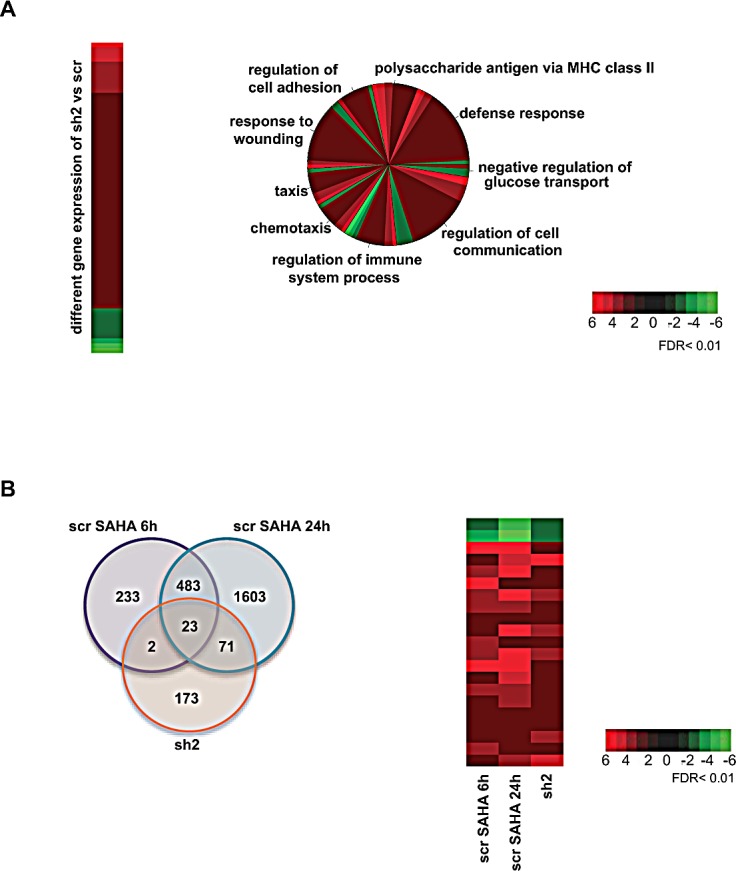
Gene expression profile A. Left panel: Microarray heat map of the 269 differentially expressed genes upon HDAC2 silencing with FDR <0.01 and fold change >2. Right panel: Pie chart showing biological processes based on Gene Ontology terms of the 269 differentially expressed genes upon HDAC2 silencing. For each gene associated with the biological processes, fold change abundance was represented by a different color gradient: red indicates upregulation; green indicates downregulation. B. Left panel: Venn diagram of the intersection between differentially expressed genes upon 6 and 24 hours SAHA treatment and HDAC2 silencing with FDR <0.01 and fold change >2. Similarly regulated genes are shown in red. Right panel: Heat map showing the expression fold change of the 23 genes in common upon 6 and 24 hours SAHA treatment and in untreated HDAC2-silenced cells: red indicates upregulation; green indicates downregulation.

To evaluate the transcriptional impact of HDAC2 silencing, the gene expression profiles of sh2 and scr clones were analyzed. T-test analysis revealed the presence of 269 differentially expressed genes by applying a high threshold of significance with a fold change >2 and an FDR <0.01. Statistical analysis showed that about 80% of the altered genes were upregulated when HDAC2 was silenced (Figure 3A). Validation of the regulation of some targets was performed and in each case corroborated the data shown (Supplementary Table 1). Gene Ontology analysis identified different cellular processes caused by changes in gene expression following silencing of HDAC2 (Figure 3A). In particular, genes involved in regulation of the immune system such as those in MHC class II were up-regulated (Figure 3A, Supplementary Figure 2A-C and Supplementary Table 1). MHC class II genes were also shown to be modulated in MDA-MB231 cells, when silenced for HDAC2 (Supplementary Figure 2B), strongly supporting a direct and cell type-independent link between HDAC2 expression and MHC class II gene regulation.

### Comparative analysis between HDAC inhibition and HDAC2 knockdown identifies specific clusters of HDAC2-modulated genes in AML

In order to determine which genes were specifically modulated by enzymatic inhibition (enzyme function required) or by HDAC2 knockdown (HDAC2 expression required) or both, several comparative analyses were performed. By comparing scr U937 cells treated with SAHA for 6 and 24 hours with the sh2 clone expression profile (Figure 3B), a cluster of 23 commonly regulated genes is observed, strongly indicating the dependence of these genes both on HDAC inhibition (SAHA) and HDAC2 silencing (Figure 3B) in these settings. These 23 genes can therefore be considered as directly modulated by HDAC2 inhibition in response to SAHA, although it cannot be excluded that other HDACs might also play a role. In addition, 71 genes were shown to be regulated by both SAHA after 24 hours and by HDAC2 silencing (Figure 3B, Supplementary Figure 2D), likely indicating a late HDAC2-dependent response to SAHA. The fact that 173 genes are only modulated by HDAC2 silencing and not by SAHA indicates that this cluster specifically requires HDAC2 expression but not its inhibition, likely suggesting the involvement of an additional repressive HDAC2 domain in the enzymatic pocket.

**Figure 4:**
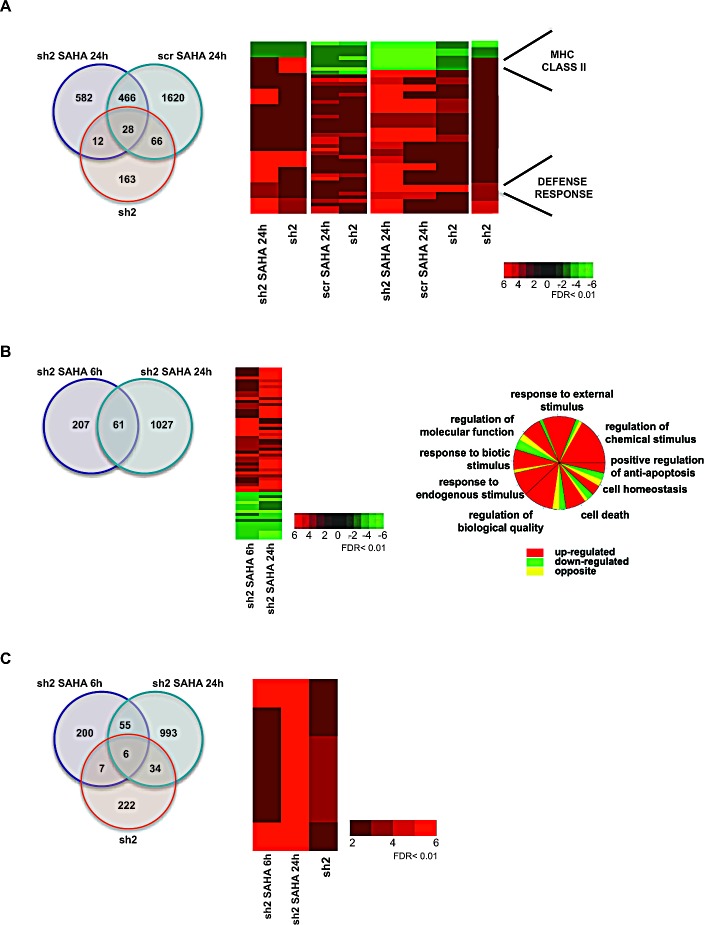
Comparative analysis between HDAC enzymatic inhibition and HDAC2 knockdown A Left panel: Venn diagram of the intersection between differentially expressed genes in sh2 clone after 24 hours SAHA treatment, in scr clone after 24 hours SAHA treatment, and in untreated sh2 clone with FDR <0.01 and fold change >2. Similarly regulated genes are shown in red. Right panel: Heat map showing the expression fold change of the 12, 28, 66 and 163 genes in the Venn diagram shown on the left: red indicates upregulation; green indicates downregulation. B. Left panel: Venn diagram of the intersection between differentially expressed genes in sh2 clone after 6 and 24 hours SAHA treatment with FDR <0.01 and fold change >2. Similarly regulated genes are shown in red. Middle panel: Heat map showing the expression fold change of the 61 genes upon 6 and 24 hours SAHA treatment in HDAC2-silenced cells: red indicates upregulation; green indicates downregulation. Right panel: Pie chart showing biological processes based on Gene Ontology terms of the 58 genes in common after 6 and 24 hours SAHA treatment in HDAC2-silenced cells: red indicates upregulated genes; green indicates downregulated genes; yellow indicates oppositely regulated genes. C. Left panel: Venn diagram of the intersection between differentially expressed genes in sh2 clone after 6 hours SAHA treatment, in sh2 clone after 24 hours SAHA treatment, and in untreated sh2 clone with FDR <0.01 and fold change >2. Similarly regulated genes are shown in red. Right panel: Heat map showing the expression fold change of the 6 genes in common upon 6 and 24 hours SAHA treatment in HDAC2-silenced cells, and in untreated HDAC2-silenced cells: red indicates upregulation; green indicates downregulation.

To corroborate and extend this hypothesis we also treated HDAC2-silenced cells with SAHA. Interestingly, when the gene expression profile induced by SAHA (24 hours) in both sh2 and scr cells was compared with that of untreated sh2 cells (Figure 4A), a cluster of 163 genes specifically dependent on HDAC2 expression (HDAC2 silencing-dependent genes) was identified. The genes in this cluster were not modulated by HDAC inhibition in either sh2 or scr U937 cells, indicating that only the silencing of HDAC2 is essential for gene expression modulation, and corroborating the fact that HDAC2-repressive action is exerted via non-enzymatic functions in these conditions. All the 163 target genes are contained in the previously identified group of 173 genes (see Figure 3B). Namely, these genes were specifically altered by HDAC2 silencing. Notably, related biological processes include primarily MHC class II antigen expression and defense response. These findings indirectly suggest that HDAC2 overexpression in cancer might be strongly related to repression of immune defense and immune recognition of tumor cells. Furthermore, the fact that a cluster of 582 genes was found to be specifically regulated by SAHA in absence of HDAC2 suggests that these genes are ‘drug responsive’ when HDAC2 is absent, indicating that gene modulation might be favored by the absence of HDAC2. In contrast, 1620 genes were exclusively regulated by SAHA in presence of HDAC2, thus being potentially dependent on HDAC2 enzymatic function alone. The 466 genes commonly regulated by SAHA in presence or absence of HDAC2 indicate that for this gene cluster HDAC2 expression is dispensable. Lastly, 106 genes were regulated by both HDAC2 inhibition (SAHA) and HDAC2 silencing.

### Impact of HDAC2 silencing on gene expression and drug response: definition of HDAC2 expression-dependent gene modulation

A Gene Ontology analysis was performed and a heat map was generated to better investigate the biological mechanisms involved after HDAC2 silencing (Figure 4B). The sh2 clone treated with SAHA for 6 and 24 hours was further analyzed, confirming that modulation of gene expression exerted by SAHA occurs when HDAC2 is silenced and that this modulation is time-dependent. Findings from these statistical tests likely reflect the fact that gene expression may be repressed by HDACs other than HDAC2 and that thus the inhibition of several HDACs by SAHA might produce similar results to HDAC2 silencing. Interestingly, 222 genes were exclusively deregulated by HDAC2 silencing and not by SAHA (Figure 4C). These 222 genes are part of the cluster of 269 genes shown in Figure 3A, corroborating and extending the finding that this group comprises HDAC2 expression-dependent genes, which are modulated only when HDAC2 is silenced, and not enzymatically inhibited, suggesting a ‘non-druggable’ HDAC2 functional repression. It should be underlined that our analysis quantitatively discriminates between gene clusters modulated by HDAC2 enzymatic function and clusters modulated by its expression.

### HDAC2 abrogates MHC class II gene expression by altering binding complexes at promoters in leukemia

**Figure 5:**
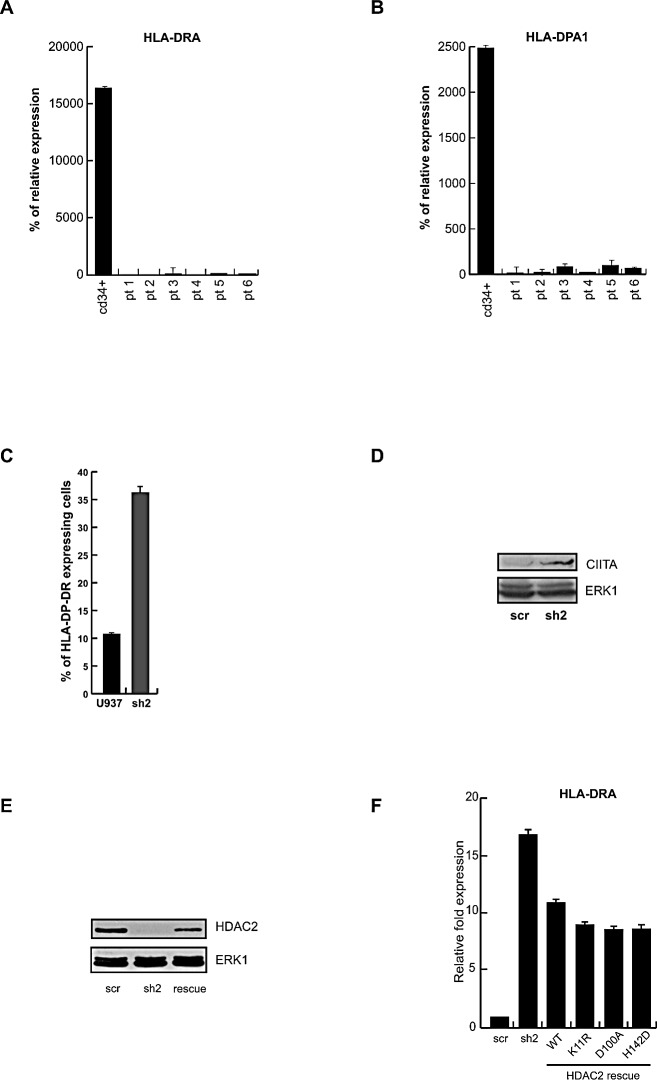
HDAC2 transcriptional impact on *HLA-DPA1* and *HLA-DRA* promoters A-B. Real-time PCR performed on *HLA-DRA* and *HLA-DPA1* genes in 6 different AML patients. CD34+ cells were used as further control. Data show mean values from three parallel experiments with error bars showing standard deviations above each column. C. HLA-DR-DP expression level measured by FACS analyses in U937 and the sh2 clone. D. CIITA protein expression levels in sh2 and scr clones. Normalization was performed with ERK1. E. HDAC2 protein expression levels in the scr, sh2 and in HDAC2 rescue clones. Normalization was performed with ERK1. HDAC2 rescue reached about 60% of the scr signal. F. Real time PCR for *HLA-DRA* gene expression in presence of scr, sh2, HDAC2 rescue (WT) and K11R, D100A, H142D HDAC2 mutants. The data show the mean values from three parallel experiments with error bars showing standard deviations above each column.

**Figure 6:**
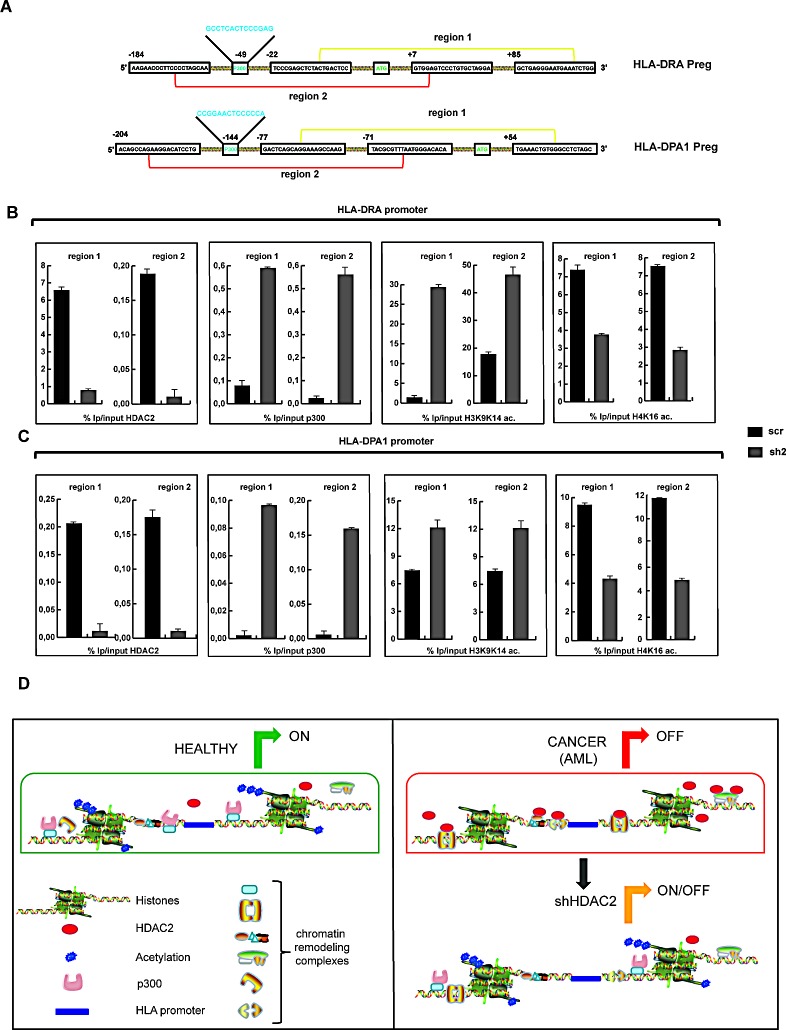
Promoter regions of *HLA-DRA* and *HLA-DPA1* genes A. Schematic representation of *HLA-DRA* and *HLA-DPA1* gene promoter regions. Region 1 (yellow) represents the part of the promoter which includes ATG region. Region 2 (red) represents the part of the promoter including a motif that recognizes p300. B. ChIP assays on *HLA-DRA* promoter in regions 1 and 2 after immunoprecipitation of sh2 and scr clones with HDAC2, p300, acetylated histone H3K9K14 and acetylated histone H4K16. C. ChIP assays on *HLA-DPA1* promoter in regions 1 and 2 after immunoprecipitation of sh2 and scr clones with HDAC2, p300 and acetylated histone H3K9K14. D. Schematic model representing HDAC2 mechanism in AML cells. Left panel*:* In healthy conditions, transcriptional machinery is active due to highly acetylated status of chromatin and low occupancy of HDAC2. Right panel: In AML, transcription is ‘off’, due to high occupancy of HDAC2 and absence of acetylation. In sh2 clone, transcription is activated by restoring very similar conditions compared to healthy state.

In order to investigate the relationship between basal expression of HDAC2 (Figure 1A) and expression of genes in MHC class II, the most represented class based on Gene Ontology analysis (Figure 3A and Figure 4A), qPCR was performed in six samples from different patients affected by AML. In these settings, the expression level of two different HLA genes, *HLA-DRA* and *HLA-DPA1* taken as examples of HDAC2 expression-dependent genes, is dramatically reduced (Figure 5A and 5B). In full agreement, HLA-DP-DR and the transactivator CIITA expression levels in HDAC2 silenced cells (sh2) were upregulated (Figure 5C-D). Interestingly, rescue experiments of HDAC2 function demonstrated that re-expression of HDAC2 in sh2 cells was able to reduce the expression of MHC class II genes (HLA-DRA) (Figure 5E). Strengthening the repressive role of HDAC2 in a non-enzymatic manner, three different HDAC2 catalytic mutants displayed similar features in these settings (Figure 5F). Given the opposite regulation of MHC class II genes and HDAC2 expression, the promoters of these two targets were selected for further investigation. A bioinformatic analysis of transcription factors (TFs) and binding site complexes of these promoters associated with potential indirect HDAC2 binding to chromatin was performed. Supplementary Table 2 and 3 contain a list of the motifs for TFs found on the promoters of these two genes which might be correlated to HDAC2. Particular attention was given to regions that may contain a p300 binding site. Figure 6A shows a schematic representation of the promoters. Region 1 corresponds to the area near ATG, while Region 2 corresponds to the area that presumably contains a p300 binding site. ChIP assays for each promoter region were performed by immunoprecipitation with HDAC2, p300, acH4K16 and acH3K9K14 (Figure 6B and 6C). Interestingly, the data obtained for both promoters show an inverse correlation between the presence of HDAC2 and acH4K16 in these areas and the recruitment of p300 with consequent increase in acetylation levels. These results corroborate and strengthen the role of HDAC2 in reducing gene expression by repressing areas of chromatin that do not allow p300 binding and consequent acetylation. Given the role of target genes, these mechanism(s) may give rise to reduced immunity surveillance within tumor cells overexpressing HDAC2. Figure 6D shows a schematic model illustrating the HDAC2 mechanism. In healthy conditions, transcriptional machinery is active due to the highly acetylated status of chromatin and low occupancy of HDAC2. Conversely, in AML, transcription is inactive as a result of HDAC2 upregulation and absence of acetylation. In sh2 clone, transcription is activated by restoring a condition very similar to the healthy state. Finally, the HDAC2-mediated mechanism of repression is one of the enzyme-independent functions exerted by deacetylases in tumorigenesis.

## DISCUSSION

Epigenetics has a key impact on the regulation of gene expression. Specifically, epigenetic modifications affect vast areas of cell biology and are involved in many human diseases. From a basic research point of view, understanding how these mechanisms are implicated in cancer and what the consequences are for tumorigenesis is crucial. Furthermore, immune system deregulation is closely associated with tumor development and maintenance. Cancer immune escape is mediated by both epigenetic events and genome aberrations involved in tumor progression. Recent findings suggest that many tumor cells do not present antigen, which drives the activation of immune response. An altered expression of MHC class II genes has been described in several hematopoietic cancers [29]. This deregulation has been correlated with highly aggressive potential, negative prognosis and loss of immune surveillance [30]. In solid tumors, however, the relationship between expression and prognosis is still unclear [31]. Some studies have reported that HDACi can turn on genes of the immune system (such as MHC class I and II genes), thus helping to recognize ‘non-self’ insults. Here, we functionally address the role of HDAC2 in AML. Although interference with HDAC2 expression in both hematological and solid cancer cells clearly demonstrates that HDAC2 exerts an anti-proliferative action, this does not seem to be its primary activity. The presence of high expression levels of other HDACs may influence the response of cancer cells to HDACi even when HDAC2 is silenced. Interestingly, the fact that the epi-mark of cancer progression [32], H4K16ac, is specifically downmodulated in HDAC2-silenced cells suggests decreased tumorigenicity, further corroborated by the lower clonal potential of these cells. This is also the case for specific chromatin areas where silencing of HDAC2 determines p300 recruitment and a general H3K9-14 acetylation, supporting a very complex chromatin remodeling with possible prioritization of acetylations. Nonetheless, the main impact of HDAC2 interference seems to be de-repression of immune functional genes. Gene expression analysis indicates that HLA complex genes are all upregulated, as are membrane proteins such as MMP-1, cadherins, cathepsins, proteins of gap junctions, integrins, proteins, and collagen, validating the hypothesis that lack of HDAC2 leads to a reorganization of complexes involved in the regulation of immune response and extracellular matrix (ECM). Following characterization of the gene expression profile induced by HDAC2 silencing, sh2 and scr clones were treated with the HDACi SAHA, which causes growth arrest and apoptosis in many tumors both *in vitro* and *in vivo*. We found a cluster of 163 genes modulated by HDAC2 silencing but not by its inhibition, clearly indicating that the functions performed by HDAC2 (and possibly other HDACs) are not purely enzymatic. In agreement, HDAC2 rescue experiments in silenced HDAC2 cells (sh2) using different catalytic mutants strongly supported that the HDAC2 repressive role on immune function is not (or not only) mediated by its enzymatic activity. Our investigation identified genes selectively regulated by the action of HDAC2. For example, the MHC class II genes *HLA-DRA* and *HLA-DPA1*, located on chromosome 6, have been labeled as targets of HDAC2. The MHC class II transactivator CIITA is a key mediator of many immunological processes through the transcriptional regulation of interferon gamma. The transcriptional activity of CIITA is regulated by several post-transcriptional modifications and, in particular, interacts with HDAC2. HDAC2 antagonizes the activity of CIITA by committing it to protein degradation. Furthermore, CIITA is considered to be the main regulator of MHC class II and therefore of immune response. CIITA is also involved in the modulation of a series of genes including *IL-4*, *IL-10*, *MMP9* and collagen type I, underscoring its importance in immune response and in the restructuring of ECM [33]. The investigation of responsive promoters further supports the immune-modulatory effect of HDAC2 silencing. In AML, the promoter regions of both *HLA-DRA* and *HLA-DPA1* display low acetylation (H3K9-14), presence of HDAC2 and absence of HATs such as p300, which has a predicted binding. Upon HDAC2 silencing, the scenario is partially reverted, displaying hyperacetylation, presence of p300 and reduced HDAC2 occupancy. Whether this effect is intrinsically correlated with the overexpression of HDAC2 in cancer, leading to an aberrant alteration of immune response or, conversely, is also present in normal cells in specific settings, remains to be determined. This is by no means a trivial question. One possible scenario is that cancer cells might ‘learn’ from normal cells, mimicking a normal and likely transitory repressive regulation possibly exerted by HDAC2 on its MHC targets during the life span of normal cells. HDAC2 overexpression may therefore play a crucial role in tumor immune escape. It is tempting to speculate that HDAC2 (and possibly other HDACs) may act as a connecting bridge between immune response modulation and cancer development. If this was the case, an opposite regulation should also apply to autoimmune disorders. Lastly, it has also been proposed that HDACi modulate MHC class I and II genes, and that this action may intrinsically contribute to their anticancer properties [34]. The hypothesis that the effect of HDACi against cancer rely on unimpaired immune capabilities, together with our findings that HDAC2 overexpression in AML leads to repression of MHC class II genes, strongly indicates that levels of HDACs and in particular of HDAC2 might impact on HDACi response *in vivo* and should be taken into consideration as a cause of possible resistance. Immune stimulatory approaches might be beneficial in these settings.

## MATERIAL AND METHODS

### Cell culture

U937 cells were obtained from ATCC. The cells were grown at 37°C in a humidified atmosphere containing 5% CO_2_ in RPMI-1640 medium (Sigma-Aldrich) supplemented with 10% heat-inactivated fetal bovine serum (FBS) (HyClone Laboratories), 100 units/mL penicillin G (EuroClone), 100 μg/mL streptomycin (EuroClone), 2 mM L-glutamine (EuroClone), 250 mg/mL amphotericin B (EuroClone) and 50 mg/mL G-418 sulfate (Invitrogen). The estrogen-independent MDA-MB231 human breast cancer cell line was cultured in Dulbecco's modified Eagle's medium (DMEM) (Sigma) supplemented with 10% FBS (HyClone Laboratories), 100 μg/mL penicillin-streptomycin solution (EuroClone), 2 mM L-glutamine (EuroClone), 250 mg/mL amphotericin B (EuroClone) and 0.5 mg/mL puromycin (Invitrogen) in a humidified atmosphere of 5% CO_2_ in air.

### Drugs

MS-275 (Bayer-Schering AG) and SAHA (Merck) were dissolved in dimethyl sulfoxide (Sigma) and used at the final concentration of 5 μM.

### Cell proliferation analysis with trypan blue

The analysis was performed using colorimetric method. The cells (2 × 10^5^ cells/mL) were plated in 6-well multi-wells in triplicate. Following stimulation at different times and concentrations (as indicated), cells were then diluted 1:1 in trypan blue (Sigma) and counted by light microscopy to distinguish dead cells (blue) from living cells, which do not stain.

### Colony forming cell assay

Supernatants in three protocols were centrifuged and re-suspended in RPMI with 10% FBS at a concentration of 5 × 10^5^ cells per mL. Subsequently, 0.3 mL of this cell suspension was added to 3 mL Metho-Cult (H4535, STEMCELL Technologies), followed by vortexing to mix thoroughly. Mixture was then kept still for 2-5 min before 1.1 mL was added to each of two or three 35 mm dishes. All cells were incubated at 37°C, 5% CO2, with ≥95% humidity for 14 to 18 days.

### Flow cytometry

Cells were harvested, washed by PBS with 1% BSA, incubated with 10μL of monoclonal anti-HLA-DR-DP-FITC antibody (Sigma-Aldrich) at room temperature for 30 min as previous described. Cells were fixed by PBS with 2% paraformaldehyde and then analysed by FACS-Calibur (BD Biosciences, San Jose, CA, USA) with the Cellquest software (BD Biosciences).

### Real-time cell proliferation

Tumor cell proliferation was monitored with the xCELLigence system (Roche). MDA-MB231 cells were suspended in DMEM and added to a 96-well microtiter plate that is specifically designed to measure cellular impedance (E-Plate, Roche, Mannheim, Germany). The measured impedance, which is dependent on the level of confluence, was expressed as an arbitrary unit called Cell Index (CI). The Cell Index at each time point is defined as (Rn-Rb)/(15X), where Rn is the cell-electrode impedance of the well when it contains cells and Rb is the background impedance of the well with the medium alone. xCELLigence monitors cellular events in real time measuring electrical impedance across inter-digitized micro-electrodes integrated on the bottom of tissue culture E-Plates. The impedance measurement provides quantitative information about the biological status of the cells, including cell number, viability and morphology. A dimensionless parameter called Cell Index (CI) is derived as a relative change in measured electrical impedance to reflect the integrated cellular status in the culture. For experiments, both scramble (scr) MDA-MB231 clone, containing the empty vector, and the sh2 clone were starved in DMEM supplemented with 10% FBS overnight before being seeded on an E-Plate 96. Two hours after seeding, scalar cell concentrations were added in triplicate. Dynamic CI values were monitored at 30-minute intervals from the time of plating until the end of the experiment. CI values were calculated and plotted on the graph. Standard deviation of tetraplicate wells for the two cells types with different treatments were analyzed using RTCA Software.

### Cell migration assay

Kinetic information about cell migration was obtained in real time without exogenous labels using the Roche xCELLigence Real-Time Cell Analyzer (RTCA) DP instrument. The RTCA DP instrument uses the CIM (cellular invasion/migration)-Plate 16 featuring microelectronic sensors integrated into the underside of the micro-porous polyethylene terephthalate (PET) membrane of a Boyden-like chamber. In this way cells migrate from the upper chamber through the membrane into the bottom chamber in response to the chemo-attractant (FBS) thus contacting and adhering to the electronic sensors on the underside of the membrane, resulting in an increase in impedance. The impedance increase is proportional to increasing numbers of migrated cells on the underside of the membrane. Moreover, CI values reflecting impedance changes are recorded by the RTCA DP instrument. Serum-free medium was placed in the top chamber to hydrate and pre-incubate the membrane for one hour in the CO_2_ incubator at 37°C before obtaining a background measurement. MDA-MB231 cells were re-suspended at the indicated concentration in serum-free medium. Once the CIM-Plate equilibrated, it was placed in the RTCA DP station and the background cell index values were measured. The CIM-Plate was then removed from the RTCA DP station and cells were added to the top chamber at the desired concentration. The CIM-Plate was placed in the RTCA DP station and migration was monitored every two minutes for several hours. MDA-MB 231 cells were analyzed in absence or presence of 10% FBS in the bottom chamber. Cell migration was detected by automated real-time monitoring and low and high seeding densities were quantitatively monitored and reflected by the CI values.

### RNA extraction

RNA extraction was performed using RNase-free material and solutions prepared with diethyl pyrocarbonate (DEPC) (Sigma) to prevent RNA degradation by ribonuclease. Cell lysis was obtained by TRIzol method (Invitrogen) using 1 mL TRIzol/10^7^ cells, according to protocol. After centrifugation at 12000 rpm for 15 minutes, Bromo-1-chloro-3-propane was added at a ratio of 1:10 with TRIzol. Once RNA was recovered, it was precipitated in isopropanol at −80°C for 30 minutes. The RNA samples were then centrifuged at 12000 rpm for 10 minutes and washed in cold 75% ethanol. Finally, they were dried at 42°C and suspended in DEPC H_2_ O. For mRNA expression, total RNA (1 μg) was reverse transcribed using SuperScript VILO cDNA synthesis kit (Invitrogen) according to the manufacturer's instructions. Primers used for qRT-PCR and semi-quantitative PCR analysis were: *HDAC2* FW: 5′-TGGTGTCAGATGCAAGCTA-3′; *HDAC2* REV:5′-TTCACCACTGTTGTCCTTGG-3′; *HLA-DPA1* FW; 5′-TGGCTGACTGAATTGCTGAC-3′; *HLA-DPA1* REV: 5′ TGAGGGGTTCTTCAAAGGAG-3′; *HLA-DRA* FW:5′-GCCCTGTGGAACTGAGAGAG-3′; *HLA-DRA* REV: 5′-CAGGAAGGGGAGATAGTGGA-3′; *HLA-DOA* FW:5′-CAGGGAGGCTGTCTTTTCTG-3′; *HLA-DOA* REV:5′-CATGATGAAACCCCGTCTCT-3′; *HLA DPB1* FW: 5′-AGTCCGATGGTTCCTGAATG-3′; *HLA DPB1* REV:5′-AATGTCTTACTCCGGGCAGA-3′. Data were normalized to the housekeeping gene *GAPDH* as follows: GAPDH FW: 5′-ATCTCCTGGCTCCTGGCA-3′; *GAPDH* REV: 5-GCTGGATGGAATGAAAGG-3′.

### Western blot analysis

After removal of the culture medium, the cells were washed with cold 1X PBS and were lysed using a lysis buffer supplemented with protease and phosphatase inhibitors: 50 mM Tris-HCl pH 8.0, 150 mM NaCl, 1% NP40, 10 mM sodium fluoride, 0.1 mM sodium orthovanadate, 40 mg/mL phenylmethylsulfonyl fluoride (PMSF), 20 g/mL aprotinin, 20 mg/ml leupeptin, 2 mg/mL antipain, 10 mM p-nitrophenyl phosphate, 10 mg/mL pepstatin A and 20 nM okadaic acid. Cells were then centrifuged at 13000 rpm for 15 minutes at 4°C, and protein content of supernatant was used to determine the protein concentration by colorimetric assay (Biorad, Italy). Cell extracts were diluted 1:1 in sample buffer 2X Laemmli (0.217 M Tris-HCl pH 8.0, 52.17% SDS, 17.4% glycerol, 0.026% bromo-phenol blue, 8.7% beta-mercapto-ethanol), and then boiled for 3 minutes. Equal amounts of protein (50 μg) were run and separated by SDS-PAGE gel (acrylamide gel). Primary antibodies used were HDAC1 (Santa Cruz), HDAC2 (Alexis), HDAC3 (Sigma), CIITA (Abcam), all diluted 1:500; 100mg/ml anti-ERK1 antibody (Santa Cruz Biotechnology) was used for normalization.

### Histone extraction

Cells were harvested and washed twice with cold 1X PBS and lysed in Triton extraction buffer (TEB: PBS containing 0.5% Triton X-100 (v/v), 2 mM PMSF, 0.02% (w/v) NaN_3_) at a cellular density of 10^7^ cells per mL for 10 minutes on ice, with gentle stirring. After a brief centrifugation at 2000 rpm at 4°C, the supernatant was removed and the pellet was washed in half the volume of TEB and centrifuged as before. The pellet was suspended in 0.2 M HCl at a cell density of 4 × 10^7^ cells per mL and acid extraction was left to proceed overnight at 4°C on a rolling table. Next, the samples were centrifuged at 2000 rpm for 10 minutes at 4°C, the supernatant was removed and protein content was determined using the Bradford assay. Antibodies against acetylated histones H3 and H4 and all hyperacetylated forms (Upstate Biotechnologies) at concentrations of 2 mg/mL were used.

### Stable transfection of the sh2 vector in U937 and MDA-MB231 cells

Silencing of HDAC2 was performed using the Sure Silencing™ sh2 plasmid vector (SuperArray Bioscience Corporation for U937 and Sigma for MDA-MB231). We tested four different sh2 nucleotide sequences able to recognize the mRNA coding for HDAC2 and induce gene silencing. The sequences were first submitted to a search by BLAST algorithm against the entire human genome sequence to ensure that only the gene of interest was recognized and silenced. In stable transfection experiments U937 cells were used at a concentration of 1 × 10^6^ per mL and were stably transfected by nucleofection using the Amaxa® Cell line Nucleofector® (Kit C for U937 cells, ATCC; Kit V for MDA-MB231 cells, ATCC). A suspension of 1 × 10^6^ U937 cells was pelleted at 700 rpm for 7 minutes, and all the medium was removed. A 12-multiwell plate with 1 mL RPMI culture medium supplemented with 10% FBS to each well was prepared and placed in the incubator at 37°C and 5% CO_2_. A mixture of 90 μL Nucleofector Solution and 20 μL Supplement for each test point was prepared. Finally, 1 μg of the sh2 vector, 1 μg of the empty vector, consisting of the negative control vector without insert for sh2 and the positive control pmax-GFP, were added at each test point. The samples were introduced into cuvettes, placed in the electroporator and pulsed with the optimized program for U937 cells. After electroporation, 500 microliters of RPMI with 10% FBS were added to each sample, which was then transferred to the previously prepared multi-well and incubated at 37°C and 5% CO_2_. After 24 hours, transfection efficiency was verified with flow cytometry by measuring the fluorescence emission of GFP positive control.

### Generation of HDAC2 catalytic inactive mutants and HDAC2 rescue in sh2 cells

HDAC2 expression vector was constructed by cloning the HDAC2 cDNA into a pcDNA 3.1/V5-His A vector (Invitrogen). HDAC2 sequence used in the rescue experiments was the following: ATCAACCCAGCGCTGTTGTTTTACAG. In addition, rescued-HDAC2 catalytic mutants expressing HDAC2^A100^ [35], HDAC2^D142^, HDAC2^R11^ were generated by using the QuickChange Lightning Site-Directed Mutagenesis kit (Stratagene) according to the manufacturers' protocol. Transfections of sh2 clones with rescued and HDAC2 mutants, were performed as previously described.

### Chromatin immunoprecipitation (ChIP)

U937 cells were diluted to a concentration of 2 × 10^5^ cells/mL the day before the experiment (usually 50 mL per flask). Cells were then cooled by placing the flask in ice. Crosslinking was performed at room temperature for 10 minutes by adding formaldehyde to a final concentration of 1%. The action of formaldehyde was neutralized by adding glycine to a final concentration of 125 mM. Cell suspension was then centrifuged at 1200 rpm for 5-10 minutes (working on ice with cold buffers and protease inhibitors). The cell sediment (pellet) was suspended in 1X PBS. Subsequently, cells were suspended in lysis buffer (approximately 20 mL per 5 × 10^6^ cells) and placed on a shaker at 4ºC for 10 minutes. Another centrifugation was performed to collect the nuclei. Immediately after the preparation of cells, or after thawing, the pellet was suspended in RIPA buffer, again in ice, at a concentration of 20 or 25 × 10^6^ cells in 500 μL of equivalent volume. The nuclei were sonicated at maximum intensity. After sonication, samples were centrifuged at 13000 rpm for 20 minutes at 4ºC. The supernatant was the extract purified and used for ChIP assay analysis. Before the sample was incubated with antibody, 10% of the sample was taken as an input indicator for further PCR analysis. The supernatant was transferred to a clean tube where the antibody was also added (about 3 μg per reaction). Immunoprecipitation was continued overnight at 4ºC with shaking and by adding 40 μL of salmon sperm DNA/Protein A agarose/BSA. The following day, the fragments were recovered by centrifugation at 1200 rpm for 5 minutes at 4ºC. The supernatant was removed with a first wash. Depending on antibody used, several washes of 3-5 minutes at 4ºC, using 500 μL of each wash buffer, were then performed. After the final wash, most of the liquid from the debris was removed and 250 μL of elution buffer was added. Elution was carried out for 30 minutes at room temperature with agitation. The fragments were sedimented and the supernatant was put into a new tube. In addition, 500 μL of elution buffer was added to the frozen input sample. Subsequently, 20 μL of 5 M NaCl was added to 500 μL of the sample. De-crosslinking was continued from 4 hours to overnight at 65ºC. Proteins were degraded by treatment with proteinase K, performed by incubating proteins with 10 μL 0.5 M ethylendiaminetetraacetic acid, 20 μL 1 M Tris pH 6.5 and 2 μL proteinase K for 1 hour at 45ºC. DNA was then recovered with phenol/chloroform, chloroform extraction and ethanol precipitation. The DNA pellet was suspended in 30-40 μL of Milli-Q water. Real-time PCR analysis was then performed on these samples. The antibodies used for this assay were p300, HDAC2 (Abcam), acH4K16 (Abcam) and acH3K9K14 (Diagenode). The following promoters were used: *HLA-DRA* promoter region 1 FW 5′-TCCGAGCTCTACTGACTCC-3′; REV 5′-CCAGATTTCATTCCCTCAGC-3′; *HLA-DRA* promoter region 2 FW 5′-AAGAACCCTTCCCCTAGCAA-3′; REV 5′-TCCTAGCACAGGGACTCCAC-3′; *HLA-DPA1* promoter region 1 FW 5′-GACTCAGCAGGAAAGCCAAG-3′; REV 5′-GCTAGAGGCCCACAGTTTCA-3′; *HLA-DPA1* promoter region 2 FW 5′-TACGCGTTTAATGGGACACA-3′; REV 5′-CAGGATGTCCTTCTGGCTGT-3′; *GAPDH* promoter FW 5′-GCTGGATGGAATGAAAGGCACAC-3′; REV 5′-ATCTCCTGGCTCCTGGCATCTC-3′.

### Microarray analysis

Microarray quality control reports generated by the Agilent Feature Extraction software were used to detect hybridization artifacts. Probe level raw intensity data were processed using R/Bio-Conductor [36] and Limma [37] packages. Background correction was performed using Limma's normexp method and data normalization was carried out in two steps: within-array loess normalization to correct systematic dye-bias and between-array quantile normalization to detect systematic non-biological bias. Ratios representing relative target mRNA intensities compared to control RNA probe signals were derived from normalized data. Differentially expressed genes between conditions (sh2 vs scr) were identified using a paired Bayesian T-test [38]. For each p-value, the Benjamini-Hochberg procedure was used to calculate the false discovery rate (FDR) in order to avoid the problem of multiple testing. The selected gene list was obtained using the following thresholds: FDR <0.01 and fold change >2. The relative abundance of biological processes based on Gene Ontology terms in each of the selected lists was analyzed using the Database for Annotation, Visualization and Integrated Discovery (DAVID) Functional Annotation Clustering tool. Genome coordinates (hg18 build) for each gene were obtained from UCSC Genome Browser. A custom bioconductor annotation package for the Agilent microarray platform was built with the AnnotationDbi Bioconductor package and used to create the chromosome plot highlighting the physical positions of genes belonging to each list with Bioconductor package geneplotter. Raw and normalized data were uploaded to the NCBI Gene Expression Omnibus (GEO) website and are accessible through GEO Series accession number GSE37529 (http://www.ncbi.nlm.nih.gov/geo/query/acc.cgi?acc=GSE37529).

### Bioinformatic analysis of transcription factors

The identification of TF binding sites was performed using Match [39], a weight matrix-based tool that uses the matrix library collected in TRANSFAC [40].

## SUPPLEMENTARY MATERIAL AND FIGURES


