# Supplementary material for: HDAC2 deregulation in tumorigenesis is causally connected to repression of immune modulation and defense escape
Source: Oncotarget. 2014 Nov 25;6(2):886–901. doi: 10.18632/oncotarget.2816 (PMC4359263; doi:10.18632/oncotarget.2816)
Supplement: Supplementary file 1 [file oncotarget-06-886-s001.pdf]

HDAC2 deregulation in tumorigenesis is causally connected to repression of immune modulation and defense escape

Supplementary Material

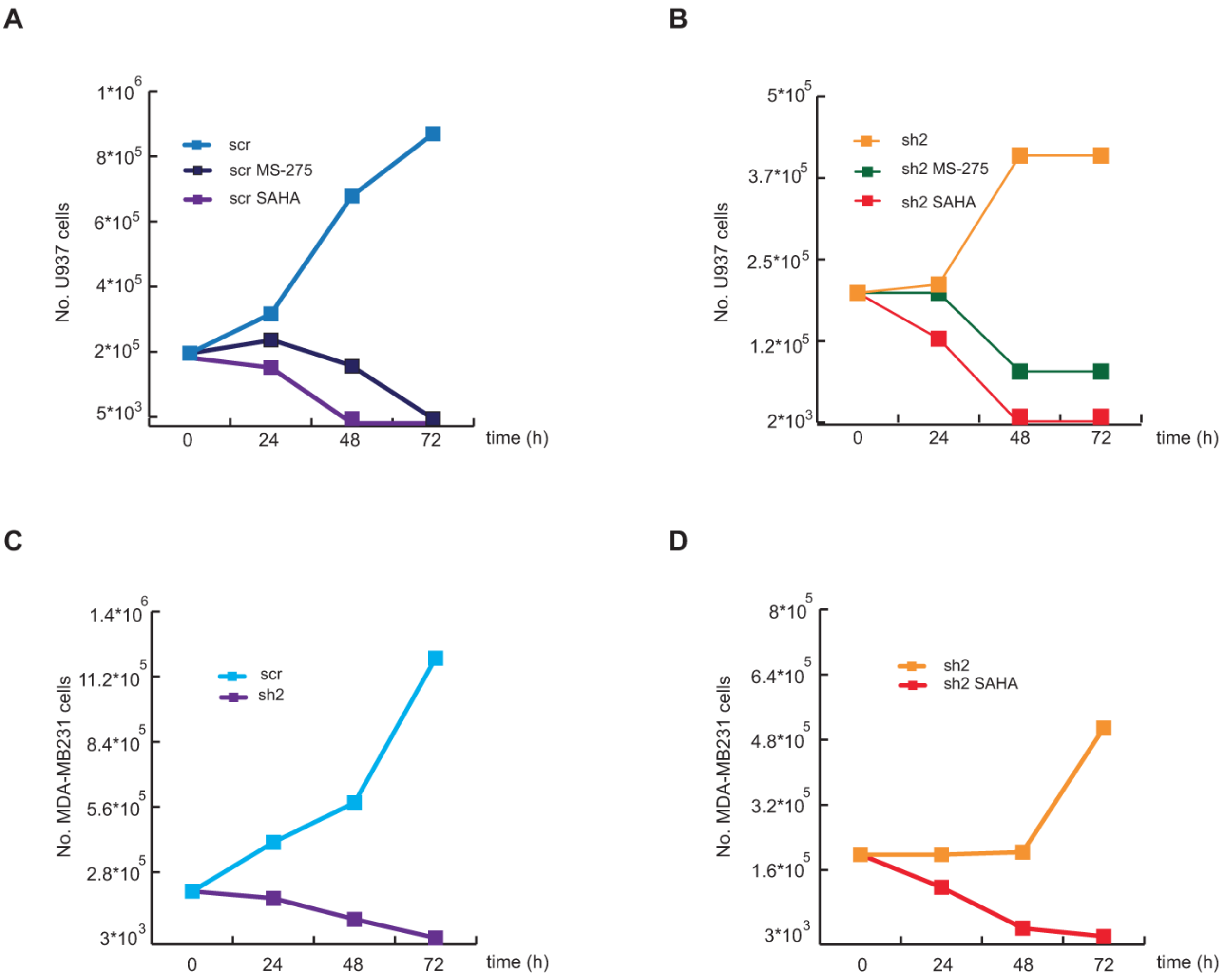

Supplementary figure 1: Proliferation rate in U937 and MDA-MB231 cell lines

A: Proliferation rate of U937 cells comparing scr clone untreated and treated with HDACi SAHA and MS-275 after 24, 48 and 72 hours.

B: Proliferation rate of U937 cells comparing sh2 clone untreated and treated with HDACi SAHA and MS-275 after 24, 48 and 72 hours.

C: Proliferation rate of MDA-MB231 cells comparing scr clone untreated and treated with HDACi SAHA after 24, 48 and 72 hours.

D: Proliferation rate of MDA-MB231 cells comparing sh2 clone untreated and treated with HDACi SAHA after 24, 48 and 72 hours.

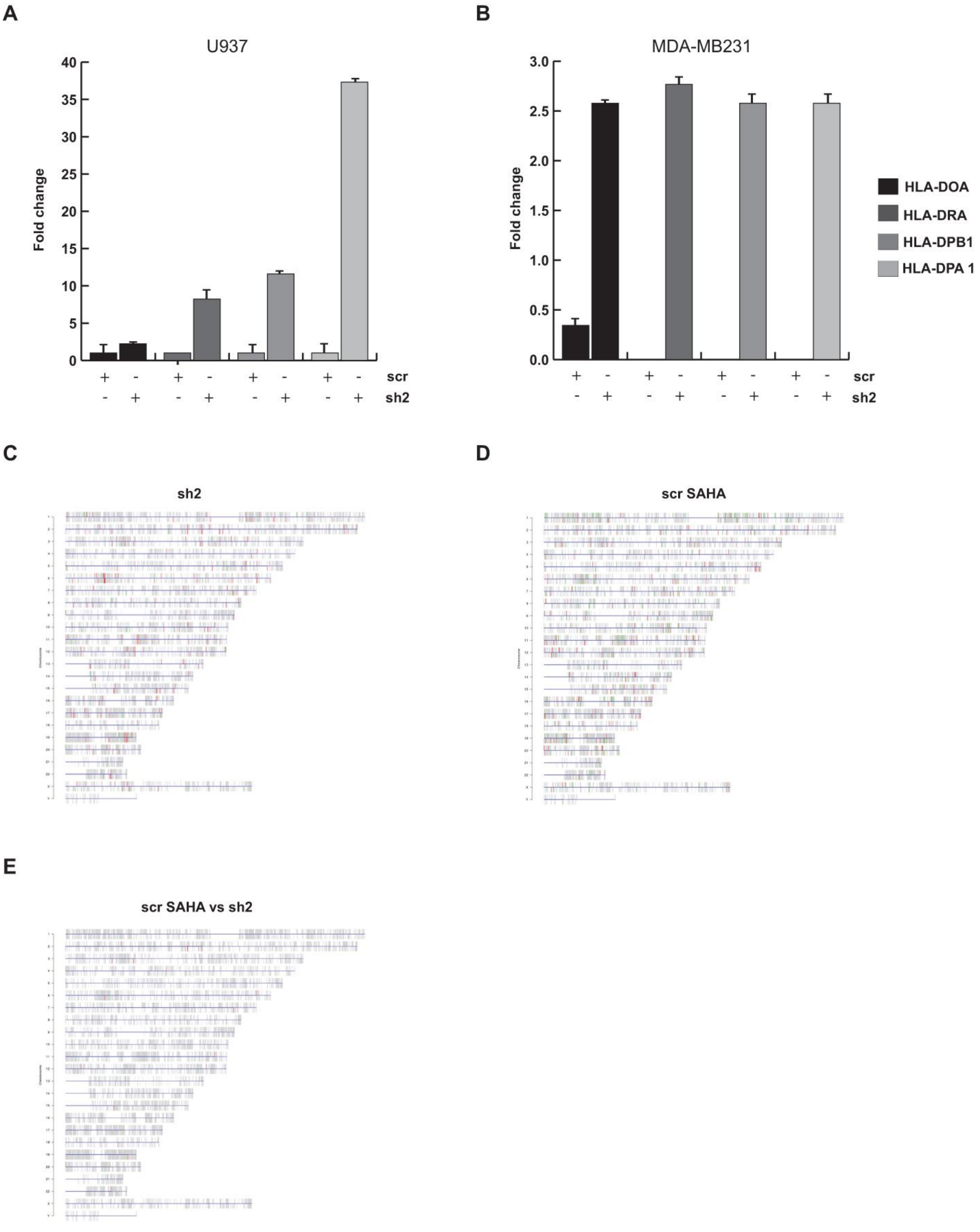

Supplementary figure 2: Genes involved in MHC class II regions

A: Real-time PCR of *HLA-DOA*, *HLA-DRA*, *HLA-DPB1* and *HLA-DPA1* genes in sh2 and scr U937 clones.

B: Real-time PCR of *HLA-DOA*, *HLA-DRA*, *HLA-DPB1* and *HLA-DPA1* genes in sh2 and scr MDA-MB231 clones.

C: Chromosome localization of the 269 differentially expressed genes upon HDAC2 silencing in U937 cells with FDR <0.01 and fold change >2.

D: Chromosome localization of the differentially expressed genes in common upon 6 and 24 hours SAHA treatment in U937 cells with FDR <0.01 and fold change >2.

E: Chromosome localization of the differentially expressed genes in common upon 6 and 24 hours SAHA treatment in U937 HDAC2-silenced cells with FDR <0.01 and fold change >2.

Supplementary table 1: Gene targets deregulated after HDAC2 knockdown

Validation of chosen gene targets deregulated after HDAC2 knockdown with relative fold changes. Genes downregulated and upregulated upon HDAC2 silencing are highlighted in bold.

Gene name	sh2
HDAC2	0,274047903
**ACT18**	**0.943765278**
**TERT**	**0.864237657**
**EHD2**	**0.664573191**
**SNURF**	**0.002591494**
**FLJ35767**	**1.437438268**
**MMP1**	**3.369419364**
**PSD3**	**1.443929196**
**ASGR1**	**1.624504793**
**EMP1**	**1.733875127**
**HLA-DOA**	**2.233025924**
**ARGHF10L**	**1.348635052**
**C16 ORF45**	**52.14655352**
**DEFB1**	**83.45924706**
**MBNL2**	**134.7834921**
**KLK1**	**12.52292787**
**MAGED1**	**26.8341819**
**C1ORF54**	**6.776310582**
**HLA-DPB1**	**11.59957466**
**AGAP2**	**10.62580325**
**CD2**	**1.274118975**
**MFAP4**	**2.484576564**
**MYBPH**	**2.030028526**
**HLA-DPA 1**	**37.31024685**
**SYTL3**	**1.2397077**
**SUCNR1**	**1.885569072**
**TYROBP**	**3.342667836**
**CAMP**	**1.9126964**
**HLA-DRA**	**8.230613663**
**RNFT2**	**6.484520959**
**TMEM**	**1.085229372**

Supplementary table 2: *HLA-DPA1* promoter regions

HLA-DPA1 promoter regions listed on the basis of their relative position on the promoter and motifs for TFs present. The region that presumably recognizes p300 is highlighted in bold.

Gene	Rel. Position	X	Sequence	Factor.name	ABS.Start	ABS.End	Strand
HLA-DPA1	797	(-)	ggcccGCGGGgc	AP-2	33048748	33048759	-
HLA-DPA1	1112	(-)	gggGAGGGg	CKROX	33048436	33048444	-
HLA-DPA1	30	(-)	gtaattATTATtta	FOXJ2	33042412	33042425	-
HLA-DPA1	1045	(+)	ccaGGAAGcgct	GABP	33048500	33048511	-
HLA-DPA1	12	(-)	CGTGG	Kid3	33049540	33049544	-
HLA-DPA1	445	(+)	CCACC	Kid3	33049107	33049111	-
HLA-DPA1	525	(+)	CCACC	Kid3	33049027	33049031	-
HLA-DPA1	585	(-)	GGTGG	Kid3	33041866	33041870	-
HLA-DPA1	748	(-)	GGTGG	Kid3	33041703	33041707	-
HLA-DPA1	868	(+)	CCACG	Kid3	33041583	33041587	-
HLA-DPA1	994	(+)	CCACG	Kid3	33048558	33048562	-
HLA-DPA1	995	(+)	CCACC	Kid3	33041456	33041460	-
HLA-DPA1	1112	(-)	ggggaGGGGGcgga	KROX	33048431	33048444	-
HLA-DPA1	122	(-)	ttttgTGACCttctc	LXR alpha	33042319	33042333	-
HLA-DPA1	167	(-)	ttttcTGACCttccc	LXR alpha	33049375	33049389	-
HLA-DPA1	211	(+)	gctgaGGTCAcctcc	LXR alpha	33049331	33049345	-
HLA-DPA1	701	(-)	tatagTGACCaaaaa	LXR alpha	33041740	33041754	-
HLA-DPA1	852	(+)	tgcagGGTCAtgggc	LXR alpha	33048690	33048704	-
HLA-DPA1	860	(-)	tctctTGACCacgct	LXR alpha	33041581	33041595	-
HLA-DPA1	1112	(+)	gGGGAGgg	MAZ	33048437	33048444	-
**HLA-DPA1**	**983**	**(-)**	**ccggaACTCCccca**	**p300**	**33048560**	**33048573**	**-**
HLA-DPA1	573	(+)	tttttCACCCc	Pax-4	33048972	33048983	-
HLA-DPA1	746	(-)	gGGGTGgggctc	Pax-4	33041698	33041709	-
HLA-	1050	(-)	gcctcagttcctcatCAC	Pax-5	33041378	33041405	-

DPA1			TGttcctgtg				
HLA-DPA1	574	(+)	ttttTCACCccacat	SREBP	33048968	33048982	-
HLA-DPA1	574	(+)	tttttCACCCca	SREBP	33048971	33048982	-
HLA-DPA1	796	(+)	gaaGGTGTaact	TBX5	33041648	33041659	-
HLA-DPA1	1008	(-)	aaGCGCGcgaact	ZF5	33048536	33048548	-

Supplementary table 3: *HLA-DPA1* promoter regions

HLA-DRA promoter regions listed on the basis of their relative position on the promoter and motifs for TFs present. The region that presumably recognizes p300 is highlighted in bold.

Gene	Rel Position	X	Sequence	Factor.name	ABS.Start	ABS.End	Strand
HLA-DRA	329	(+)	cCAATCt	HOXA7	32406947	32406953	+
HLA-DRA	789	(-)	aGATTGg	HOXA7	32407407	32407413	+
HLA-DRA	95	(-)	GGTGG	Kid3	32406713	32406717	+
HLA-DRA	98	(-)	GGTGG	Kid3	32406716	32406720	+
HLA-DRA	190	(+)	CCACC	Kid3	32406808	32406812	+
HLA-DRA	13	(+)	cacgaGGTCAggagt	LXR alpha	32406631	32406645	+
HLA-DRA	284	(-)	ttcatTGACCtgaat	LXR alpha	32406902	32406916	+
**HLA-DRA**	**1061**	**(-)**	**gcctcACTCCcgag**	**p300**	**32407679**	**32407692**	**+**
HLA-DRA	892	(+)	atgAACGGa	v-Myb	32407510	32407518	+
